# New psychoactive substances in Eurasia: a qualitative study of people who use drugs and harm reduction services in six countries

**DOI:** 10.1186/s12954-020-00448-2

**Published:** 2020-11-30

**Authors:** Eliza Kurcevič, Rick Lines

**Affiliations:** 1 Eurasian Harm Reduction Association, Verkių g. 34B, office 701, 08221 Vilnius, Lithuania; 2 School of Law, Swansea University, Richard Price Building, Singleton Park, Swansea, SA2 8PP UK

**Keywords:** New psychoactive substances, NPS, Harm reduction, Eurasia, Belarus, Moldova, Serbia, Kazakhstan, Kyrgyzstan, Georgia

## Abstract

**Background:**

This study examines the use of new psychoactive substances (NPS) and the harm reduction response in six Eurasian countries: Belarus, Moldova, Serbia, Kazakhstan, Kyrgyzstan, and Georgia. The aim is to identify current patterns of NPS use and related harms in each country through recording the perspectives and lived experience of people who use drugs and people who provide harm reduction services in order to inform the harm reduction response.

**Methodology:**

The study involved desk-based research and semi-structured interviews/focus groups with 124 people who use drugs and 55 health and harm reduction service providers across the six countries.

**Results:**

People who use drugs in all countries were aware of NPS, primarily synthetic cathinones and synthetic cannabinoids. NPS users generally reflected two groups: those with no prior history of illicit drug use (typically younger people) and those who used NPS on an occasional or regular basis due to the lack of availability of their preferred drug (primarily opiates). In many cases, these respondents reported they would not use NPS if traditional opiates were available. Common factors for choosing NPS included cost and accessibility. Respondents in most countries described NPS markets that use the DarkNet and social media for communication, secretive methods of payment and hidden collection points. A recurring theme was the role of punitive drug policies in driving NPS use and related harms. Respondents in all countries agreed that current harm reduction services were important but needed to be enhanced and expanded in the context of NPS.

**Conclusions:**

The study identified patterns and drivers of NPS use, risk behaviours and drug-related harms. It identified gaps in the current harm reduction response, particularly the needs of non-injectors and overdose response, as well as the harmful effects of punitive drug policies. These findings may inform and improve current harm reduction services to meet the needs of people who use NPS.

## Introduction

This study examined the use of new psychoactive substances (NPSs) and the harm reduction response in six Eurasian countries—Belarus, Moldova, Serbia, Kazakhstan, Kyrgyzstan, and Georgia. The research engaged people who use drugs in order to record their lived experience of NPS use, NPS markets and NPS-related harms. Researchers also engaged with people providing harm reduction and health services, to draw out their experiences of responding to NPS use. This study represents the first detailed, multi-country investigation of NPS use and harm reduction undertaken in Eurasia, one that focusses on the community response among people who use drugs and service providers. The results supplement the scarce data on the use of NPS in these countries and the region as a whole, and may contribute to building a more accurate picture of the use of new psychoactives that can inform policy change and the harm reduction response.

The United Nations Office on Drugs and Crime defines new psychoactive substances as “substances of abuse, either in a pure form or a preparation, that are not controlled by the 1961 Convention on Narcotic Drugs or the 1971 Convention on Psychotropic Substances, but which may pose a public health threat” [1]. In other words, these are synthetic substances designed to mimic the psychoactive effects of more traditional illicit drugs. While not all new psychoactives are truly “new” substances [1], their presence in the illicit drug marketplace is an attempt to “outwit drug laws by producing drugs that are not controlled” under criminal law [2]. Some countries have criminalised new psychoactives in domestic law, even though they are not controlled under the international drug treaties [3]. In response, substances continue to have their chemical composition “tweaked” to try to skirt regulation [3], creating what has been described as a “cat-and-mouse game between legislation and clandestine laboratories…with new designer stimulants replacing those outlawed almost as soon as legislation passes” [4].

Most new psychoactives fall into two general categories. Synthetic cathinones mimic the effects of amphetamines, cocaine and ecstasy and are typically ingested orally, inhaled or injected [4, 5]. Synthetic cannabinoids mimic the effects of cannabis and are typically mixed with tobacco or other herbal mixtures and smoked [5, 6]. In Western Europe, the most popular new psychoactives are methcathinones and phenethylamines, drugs that mimic the effects of cocaine and ecstasy [7]. For this reason, discourse on NPS use in Western Europe has tended to focus on young people using new psychoactives in nightlife settings, clubs or music festivals [8–12].

Anecdotal data and reports from civil society organisations across Central and Eastern Europe and Central Asia (CEECA) collected by the Eurasian Harm Reduction Association (EHRA) indicate that NPS use is increasing in many countries in the region, particularly among people who inject drugs. With some important exceptions [4, 13], vulnerable or marginalised people who use drugs, and people who inject drugs, do not figure prominently within the Western European research on NPS use and harms. For example, of the more than 3000 NPS users surveyed in a 2019 study across six European countries (Germany, Hungary, Ireland, the Netherlands, Poland and Portugal), less than three hundred were identified as “socially marginalised users”, the vast majority of these being in Hungary and Poland, the eastern region of the European Union [11]. The anecdotal reports gathered by EHRA on increasing NPS use among people who inject appear consistent with the data drawn from Hungary and Poland in the above study [11] and is a situation also reported by other pan-European non-governmental organisations [14].

New psychoactive substances have been associated with various health harms [6]. NPS injecting has been linked with increased unsafe injecting practices [6, 13, 15, 16] and increased hepatitis C and/or HIV prevalence [17], particularly in Eastern European countries [15, 16]. Synthetic cathinones are described as producing a short-lived euphoria, leading to more frequent injecting practices [6]. It is generally assumed that most people who inject NPS have a previous history of injecting more traditional illicit drugs [6], although some research has documented people initiating injecting with NPS [13].

Harms from the use of synthetic cannabinoids are also well documented [18], including in Eastern Europe [6]. These include physical effects such as increased heart rates and blood pressure, sometimes leading to strokes or heart attacks, as well as psychological harms such as psychosis [18]. Both synthetic cathinones and synthetic cannabinoids have been linked to cases of overdose and poisoning [18–22].

These developments create new challenges for harm reduction services, which have been developed in the context of opiate injecting and the prevention of blood-borne virus transmission.

## Methods

### Study design

The study was a partnership between EHRA and the School of Law, Swansea University, UK. The six countries were selected for three reasons. (a) Feedback from communities of people who use drugs and civil society organisations in these countries indicated the need to examine NPS use. (b) The lack of comprehensive information on NPS use and related harms in each country. (c) The six countries represent four distinct Eurasian regions: Kazakhstan and Kyrgyzstan (Central Asia region); Georgia (Caucasus region); Serbia (South-Eastern Europe region); and Belarus and Moldova (Eastern Partnership neighbours).

Stage 1 of the research consisted of desk-based research to survey and collate the available national data on NPS (official reports, media, peer-reviewed publications, literature not indexed in medical databases, documents from national government and regional/international organisations). Desk-based research informed preparation of the questions for key respondents, who included people who use drugs, harm reduction service providers and medical professionals. Stage 2 consisted of semi-structured interviews and/or focus groups with key respondents and compiling and analysing the data collected.

### Data collection

EHRA is a network uniting 303 members in CEECA region, and the researchers used this network to identify and engage key respondents in each country. As the study aimed to gather information directly from people who use drugs, the researchers engaged organisations providing harm reduction services in each country. These in-country organisations assisted the researchers to recruit people who use drugs to participate. The researchers also identified, where possible, country-level organisations of key affected populations, including MSM, sex workers, people who use drugs and young people. Where such organisations existed, they were invited to participate in interviews or focus groups. Where possible, the researchers sought to organise interviews or focus groups in more than one city or town to seek a better understanding the situation for NPS use in the country.

Focus groups and interviews took place between April 2019 and June 2020. Eliza Kurcevič (EK) conducted interviews and focus groups in Kazakhstan and Kyrgyzstan. Due to the COVID-19 travel restrictions that came into force in early 2020, interviews in Kyrgyzstan were conducted online using Zoom or Skype. In Belarus, Moldova, Georgia and Serbia, the authors contracted qualified and experienced country-based researchers to conduct interviews and focus groups. These contracted researchers followed the methodology and framework established for the study and were supervised by the authors, who analysed the data collected. Interviews and focus groups were conducted in Russian, Georgian, Serbian or Moldovan (as appropriate) and were audio-recorded and later transcribed (Table 1).

**Table 1:** Respondents

Country	People who use drugs	Harm reduction/health professionals	Sites of interviews/focus groups
Belarus	11	3	Minsk and Mogilev
Moldova	12	15	Chisinau and Balti
Georgia	23	12	Tbilisi
Kazakhstan	40	15	Temirtau, Karaganda and Almaty
Kyrgyzstan	20	5	Bishkek and Osh
Serbia	18	5	Belgrade

### Ethical considerations

The Ethical Review Committee of the School of Law at Swansea University Study reviewed and approved the research methodology and safeguards. All study participants were aged 18 years or older and voluntarily agreed to take part. All were advised of the purpose of the research and what was expected from them as participants. Debriefing was conducted after each interview or focus group, including an opportunity for participants to identify concerns or areas for further inquiry.

All participants provided prior written consent. Consent forms were securely stored and an identifying code given to each participant (“Participant A”, etc.) to ensure anonymity. All participant data were stored securely and only used for the purpose of the project. The research followed the legal guidelines of the European Union General Data Protection Regulation (GDPR), and the standards of the Ethical Review Committee of the School of Law, Swansea University.

The people who use drugs who participated in the study were given the choice of whether to take part in focus groups. They were informed that their decision would have no impact on their access to support groups or harm reduction services. A member of the harm reduction service or peer group was available to provide support if required. Given the illegal status of NPS, researchers explained they would not ask details of individual NPS use but would instead talk broadly about NPS use in the wider community of people who use drugs. It was explained that all participant information would be anonymised. Member checking was carried out following the collection of the data.

## Results

### Introduction of NPS into domestic markets

According to respondents, NPS first appeared in Belarus in 2008 to 2009. Prior to that time, the main drugs consumed were opiates made from poppy seeds and poppy straws. Opiates remained the main psychoactive substances used in the early years of NPS availability. However, in January 2014, the President of the Republic of Belarus issued a decree expanding criminalisation of poppy seeds [23]. The impact was to significantly reduce the availability of traditional opiates, ushering in a significant increase in the use of new synthetic drugs that transformed the domestic drug market. By 2017, NPS occupied 40% of the illicit drug market, with opiates comprising just 10% [24].

In Moldova, respondents identified various years in which new psychoactives first appeared in the drug market. Some participants stated this occurred as early as 2010, while others identified 2013–2014 and 2015–2016. The first media reports on NPS in Moldova were published in 2014 [25, 26].

In Georgia, respondents reported that synthetic cannabinoids started to appear in 2013–2014. From 2018, the use of synthetic cathinones (“bath salts”) became widespread. Media attention in 2013–2014 led to the adoption of 2014 legislation on new psychoactive substances and to the amendment of the penal code to criminalise NPS production, purchase, storage and related activities [27]. That same year, the Ministry of Internal Affairs launched the campaign “No to New Psychoactive Drugs—Lets Change Attitude Together” [27].

In Kazakhstan, respondents reported that new psychoactives first appeared in 2009–2010. New psychoactives appeared earlier in Kazakhstan than many other countries in the region due to its borders with China and Russia, which manufacture a large proportion of NPS, which are trafficked to or through Kazakhstan.

In Kyrgyzstan, respondents reported that new psychoactives first appeared in 2013, when synthetic cannabinoids entered the drug market. This was also the first year NPS use was reported in the national media [28]. New psychoactive substances went unregulated by national laws for several years and were widely used, especially among young people. In 2015, the government adopted a law banning synthetic drugs, including “spice” and other herbal smoking mixtures. As the availability of synthetic cannabinoids started disappearing, synthetic cathinones (“salts”) appeared on the drug market.

In Serbia, respondents stated that NPS appeared in 2010–2011, but gained popularity in 2013–2014 when synthetic cannabinoids became widely available through “Smart Shops”. Synthetic cannabinoids were legal at this time and particularly popular among young people. Synthetic cathinones—specifically mephedrone (“meow meow”)—were also associated with this period and were sold both to recreational users as MDMA and to opiate users who injected it. However, after changes to legislation in 2015, the use of both synthetic cathinones and synthetic cannabinoids decreased.

### Types of NPS used and common slang names

Respondents in each country were asked to identify the most common new psychoactives in circulation, as well as the common names used for them.

**Table:** 

Country	Common NPS used and slang names
Belarus	Synthetic cathinones (“salts”) and synthetic cannabinoids (“spice”) were the most common types of new psychoactives identified. Slang names for synthetic cathinones included Alpha-PVP (available in various colours); “Sabaka” (which means “dog” in English); Mephedrone (also called “Mefer”); “Black Mamba”; and “Dosia”, “Daska”, “Kedy”, “Krasofki”, “Skorost”, and “Speed” (for amphetamine-type substances). For synthetic cannabinoids, slang names included smoking mixtures, “Ligalka”, “Liga” and “Ximlo”
Moldova	Respondents identified a number of common new psychoactives including: Mixes; Spice; Skorost (“speed” in English); Mephedrone; PVP; Energetics; Speed; JWH (a synthetic cannabinoid)
Georgia	The most frequently consumed substances were identified as Alpha-PVP, NBOMe, ketamine, synthetic cannabinoids (“spice”), mephedrone and speed. Some slang names for synthetic cathinones included bath salts; salts; crystals; Alpha-PVP (or PVP); Muka (“flour” in Russian); Speed; Flakka; Mephedrone (or “meph”). Names for hallucinogens included Mark; Blotter; NBOMe; Gin; Acid. Names for synthetic cannabinoids included Bio; Bio-marijuana; Bio-smoke; Bio-hashish; Spice; Chocolate; Cherry; Tea; Green; Black; White; Yellow
Kazakhstan	Common slang names for synthetic cathinones included salts; bath salts; SK; Skorost (speed); Red dragon; Ruby; Muka (flour); Watermelon; Crystals; Alpha-PVP; Mephedrone; Meow; 4-MMC; Meph. Common names for synthetic cannabinoids included JWH or Dzhivik; Spice; Chamomile; St. John’s Wort; Aqua; Shiza
Kyrgyzstan	Respondents reported that new psychoactives were referred to in various ways. The primary method was to describe NPS by its form: Salt (slang name: “solyaga” or “solyara”); Crystals; Flour; Sugar. The other was to call it by its chemical name: Mephedrone (or “meph” for short); Alpha-PVP; although this was less common. The most common slang to describe NPS included SK (meaning salts); Speed; Cosmos; Blue stone; Snowy flour; Rahat; High; Take-off; Chinese salts; Bath salts. The other common way was to describe NPS by colour: blue, red, white, yellow, etc.
Serbia	Respondents were not entirely clear what constituted “new” psychoactives. However, the following substances were identified by as being “new” in the context of the Serbian drug scene; GHB/GBL (“G”); Synthetic cannabinoids (“Herbal incense”, “spice/”, “Black Mamba”); 2CB; PCP/3MEOPCP; Alpha-PVP (PVP); Ketamine (K, Special K); Mephedrone (“Meow Meow”); Flex (synthetic cocaine); 25I-NBOMe

### Patterns of use

In Belarus, synthetic cathinones were becoming the primary drug injected due to accessibility and cost. However, intravenous use was more likely to occur among people who had previously injected other opioids. In Moldova, the most common methods of NPS use were smoking, injecting and inhalation. Focus group participants stated NPS was more prevalent among young men and women under the age of 35 than among other age groups. These new consumers and younger consumers predominantly smoked or inhaled NPS. In Georgia, NPS were consumed by smoking, sniffing, swallowing, injecting and sticking under or on top of the tongue (“blotters”). Putting drops in the eyes was common for hallucinogens (liquid acid). Injecting NPS use was more likely among people who had previously injected other drugs, including opioids.

In Kazakhstan, NPS were used both by people with a history of more traditional drug use and by people who started with NPS. However, the two groups usually differed in age and route of administration and did not interact with each other. Among experienced and older users, the most common routes of administration were smoking and injecting. Younger people with no previous drug use history preferred smoking and snorting as these methods were simpler and required no additional preparation or special equipment/paraphernalia. However, some respondents reported that people who started by snorting or smoking NPS sometimes began injecting after 3–4 months because of an increased tolerance.

In Kyrgyzstan, the most common methods of using NPS were smoking (through pipettes, bulbs) and snorting. Respondents reported NPS use was common among young people, including school and university students. This group was described as being afraid of injecting and preferred to use NPS in ways other than intravenously, such as by wrapping substances in paper and taking them orally (so-called “bombs”). Young people were described as having a culture of sharing information about NPS (where to purchase, which were the best, how to use, etc.). There was also a culture of using in groups, called “marathon gangs” because the group will use NPS for several days in a row.

In Serbia, the most common methods of consuming NPS were smoking, snorting and oral ingestion. NPS consumers fell into two primary groups. The first were members of the MSM community who used GHB/GBL and other substances in the context of social and sexual activities, to reduce physical and sociocultural inhibitions. All study respondents from the MSM population were active users of GHB. The second group were young people who used NPS in recreational settings, such as parties, festivals or similar events. This group was comprised both people who knowingly consumed NPS and people who believed they were taking a more traditional illicit substance such as MDMA or cocaine.

With the exception of Serbia, all the countries reported injecting NPS to be common. In all these cases, injecting was more likely to occur among people with a previous history of injecting other drugs, particularly opiates. In Moldova, for example, respondents estimated that synthetic cathinones represented more than 70% of cases of all injecting drug use, resulting in significantly reduced use of other opioids because of substitution with these new injectable substances. However, in Serbia intravenous use of NPS appeared to be very rare, and respondents reported no examples of injecting in their networks. Respondents from the MSM community observed that injecting (so-called “slamming”) was not as popular in Serbia as in some other European countries. Explanations for this included the lower purchasing power in the country and the stigma surrounding injecting drug use in Serbian society. Respondents in Serbia reported that people who inject drugs (primarily opiate users) and sex workers generally preferred to use traditional illicit substances or prescription drugs. While opiate users might consume NPS on occasion, this would typically occur on a situational basis when dealer had it available and offered it, not because the opiate users actively sought out NPS.

Smoking NPS was reported to be common in most counties among both cohorts. Almost 80% of respondents in Moldova reported using “spice” as a smoking mixture. In Georgia, the synthetic cannabinoid, “Bio”, was typically sold in powder or crystal form and mixed with tobacco and smoked. Participants reported that “Bio” had a short-term effect that usually lasted 10 min. For this reason, it could be smoked 50 or more times a day. Participants in Serbia reported that the use of synthetic cannabinoids was very popular for a period, even among people who used opiates, because they were legal and inexpensive. However, this popularity drastically diminished when synthetic cannabinoids were criminalised.

Participants in Georgia, Serbia and Kyrgyzstan identified poly-drug use as common among people who use NPS, and that new psychoactives typically were used in combination with other drugs. In Georgia, for example, common drug combinations identified included: Ketamine + Speed + Amphetamine (so-called “Trinity of Berlin”); Alpha-PVP + Bio-marijuana; Alpha-PVP + Ketamine; Speed + Ketamine; Amphetamine + Bio-marijuana; Ecstasy/MDMA + Bio-marijuana; Ecstasy/MDMA + Speed; LSD/NBOMe + Mushrooms + Bio-marijuana (so-called “Candy Flip”). In Kyrgyzstan, NPS were often used in combination with pharmaceutical drugs rather than with illicit drugs. In Serbia, NPS were commonly consumed in combination with amphetamines, cocaine, ecstasy, alcohol and cannabis. There were several reasons identified for the popularity of drug combinations: to prolong or intensify the drug’s effect; to change one drug’s effect by adding another (e.g. a stimulant effect with a hallucinogenic effect); to accelerate the effects of a drug (to reach “kick-in” sooner); or to handle coming down or reduce drug hangovers.

### Dosage and potency

In Belarus, respondents stated that synthetic cannabinoids (“spice”) had grown in potency over the past decade. One participant noted that 10 years ago, one gram of reagent could be mixed with herbs to produce seven grams of smoking mixture. Today, one gram of reagent produced 30–40 g of smoking mixture. For people who used synthetic cathinones, the daily dosage varied greatly depending on the substance, its quality and the individual’s tolerance. For example, one gram of “salts” such as “Alpha-PVP” and “Sabaka” was generally sufficient for 20–25 injections. Because two or more people typically used together, this equated to 10–15 injections per night/per person. From one gram of mephedrone, it was possible to prepare 3–4 injections. Respondents described the effects of mephedrone as short lasting, leading to a greater number and frequency of injections. Participants used the term “toler” to describe growing tolerance of NPS over the course of several days of use. For example, if a person used 0.5 g of NPS on the first day, he or she would likely need 2.5 g by the fifth day.

In Moldova, virtually all participants reported that one gram of “Bio” marijuana was enough for 100 (or even more) doses, depending on potency (black “Bio” was identified as the most potent and green “Bio” the least).

In Georgia, participants reported that one gram of synthetic cathinones prepared up to 30–40 injections, 20–25 smoking doses and 10–20 sniffing doses. Participants noted that injecting and smoked/sniffed dosing occurred every 40–60 min. NPS dosage was described as being dependent on the person’s experience and tolerance, with new or less experienced people using lower doses than more experienced users. On average, participants reported 10–15 injecting or smoking/sniffing episodes per day by an individual.

In Kazakhstan, respondents could not identify a typical daily dose. NPS tolerance was described as growing quickly, and the euphoria experienced in one dose as short lasting. As a result, NPS were commonly used until finished and a person purchased more. In Kyrgyzstan, respondents also could not identify a typical daily dosage, although most agreed that NPS were used continuously until there was none left. The effect of NPS was described as being a quick, short-lasting euphoria. If a person or group purchased a larger amount of a substance, it could be used for 5–10 days or more without breaks until it was finished.

### Reasons for choosing NPS

The low price of NPS was among the most common factors identified for the popularity of new psychoactives (Belarus, Moldova, Georgia, Kazakhstan, Kyrgyzstan), particularly among younger people unable to afford more traditional drugs. In Belarus, for example, respondents said that heroin, opium, hashish and cannabis were five to ten times higher than the price of NPS. Related to the issue of cost was that of potency. A person could obtain more doses from one gram of NPS than a gram of more traditional illicit drugs.

Along with pricing, the inaccessibility of traditional illegal drugs was the other most commonly cited reason for using NPS (in all countries other than Serbia). This was particularly the case among respondents with previous drug use histories (typically injecting drug use) who substituted new psychoactives for their preferred drug of choice due to either high cost or general lack of availability in the market. In Moldova, respondents stated NPS were used as a temporary substitute for illicit drugs such as opium or heroin that were not accessible. Respondents in Kazakhstan and Kyrgyzstan described heroin as disappearing from the drug market, and NPS use occurred because people lacked other options. Respondents in Belarus similarly reported they would prefer to use traditional substances if they were available. In Kyrgyzstan, most respondents stated that they would never have started using NPS if opiates had been available on the market.

In Georgia and Kyrgyzstan, sex workers interviewed stated that NPS helped them with work performance (i.e. to work longer hours) and to reduce fear and anxiety. In Serbia, sex workers generally did not have much experience with NPS and preferred to use traditional illicit substances or prescription drugs. Members of the MSM community interviewed in Serbia, Kyrgyzstan and Kazakhstan described using NPS in the context of sexual activities, to reduce physical and sociocultural inhibitions and to be part of the cultural identity of the community, which is also documented elsewhere in the literature in Europe [29]. Some respondents in Belarus, Moldova and Kazakhstan noted NPS were more difficult to detect in urine tests and therefore there was a reduced risk of being penalised for their use, such as sanctions for people currently on opioid substitution treatment (OST) programmes.

### NPS markets, purchasing and distribution networks

Respondents in most countries (Belarus, Moldova, Georgia, Kazakhstan and Kyrgyzstan) described new psychoactives as being easy to access. NPS purchase and distribution commonly took place via the use of websites, social media platforms and various messaging apps.

In Belarus, for example, people primarily accessed NPS via DarkNet drug markets (“Hydra”, “Koncern Kalashnikov” and “Zubr”) or through social media platforms. Participants sometimes received random messages on social media about purchasing NPS, or were added into temporary networking groups/pages. NPS websites were also advertised in the streets of Belarus by painting website addresses on walls. Once placing an order, the customer transferred funds to the administrator’s bank account, usually via ATMs. After payment, a photograph of the terminal screen or of the paper receipt served as confirmation. Shortly after confirming payment, the customer received an address with a photograph of a hiding place where the substance could be collected. Sometimes the seller also sent GPS coordinates of the pickup location. Respondents noted that it took 1–5 h to receive NPS purchased through this method. It was also possible to purchase so-called constructors, legal chemical components from which people may themselves make potent NPS using step-by-step instructions provided by the seller.

The procedure described in Belarus was common in most other countries examined. In Moldova, NPS sales took place through social networks such as *Odnoklassniki* and *Instagram*, or via fake Facebook pages with hidden IP addresses. These networks published a list of substances and prices called “offers”. There were also random messages sent to people via social networks, with the intention that these messages would be circulated and eventually reach interested customers who would then circulate them further. The *Telegram* application was widely used to sell NPS. These limited-access chat rooms or groups brought together NPS sellers and customers to share prices and types of NPS on the market. Once a potential customer specified their location, the substance desired and quantity requested, the money transfer and delivery were made using the same procedure described above.

In Georgia, new psychoactives were mainly sold through online drug markets such as *Matanga* and *Party Doc*, as well as through messaging apps such as *Telegram*, *Viber* and *WhatsApp*. Messages were sometimes sent randomly to people on social media apps, especially on *Viber*, mostly in Russian language, with the aim of eventually reaching interested customers. The practice of direct hand-to-hand purchases of NPS was also common in Georgia. In such cases, middle agents (“legs”) played the main role, as this was considered a safer way of buying NPS than via online markets. Respondents reported that this distribution method was typically based around friendships and operated within informal social networks.

In Kazakhstan, the most common purchase method was through *Telegram* or *WhatsApp*, using an electronic wallet (“kiwi”) to make payments and “zakladki”, the hidden packages delivery system reported in other countries in this study. Buying NPS online included a number of different actors playing roles in the process. In a best-case scenario, the transaction included four or five actors: (1) the Customer (2) the Administrator of the bot, who referred the Customer to (3) the Operator, with whom the Customer made the deal. As soon as payment was made, (4) the “Prikopper” (“zakladchik”) hid the package and the Customer received GPS coordinates and/or a photograph of the hiding place. The Customer then either collected the package himself or herself, or asked (5) a “Toptun” (so-called “legs”) to collect the hidden package on behalf of the Customer, in return for a share of drugs.

Other actors could also come into play. There were “Shkurohody”, people who knew the locations of the usual hiding places, and could hunt for packages. In cases where neither the Customer nor the “Toptun” was able to find the hidden package, they could contact the “Equal Shop”, which acted as a sort of customer claim centre. There were also so-called “Collectors”, people who tracked down unreliable customers or people who stole hidden packages. NPS were also sold face-to-face through dealers, or face-to-face with minimal human interaction, for example, by delivering the NPS and leaving it hidden close to customer’s home. If a customer had a good relationship with a seller, he or she could request that substances be brought to their home and left in a discreet place, such as a stairwell. In some cases, it was possible to acquire NPS without money, in exchange for sexual services in exchange or by working as a “zakladchiki” (courier) paid with NPS.

In Kyrgyzstan, NPS were primarily purchased online. Respondents described extensive and aggressive marketing of NPS through various means, including *Telegram* and advertising signs posted on walls. Young people were also recruited as “advertising agents” to distribute signs and posters around the city and to act as couriers to deliver the purchases to agreed hidden locations where the customers could collect them. There were risks identified with online purchasing. These included the possibility of receiving a different substance than that ordered, not receiving any substance at all if an online store was fake, having the NPS stolen from the hiding place or being arrested while collecting the hidden package. NPS were also available through a “hut”, a house or flat where people could purchase and use small, single-dose quantities if they lack the funds or knowledge to make internet purchases.

In Serbia, different groups of consumers purchased NPS in different ways. The MSM population, which primarily used GHB, purchased through dealers, in some cases dealers promoted via dating apps used by the MSM population such as *Grinder*. The customer called or messaged the seller, and the substance was delivered a few hours later to the customer’s address, where payment was made. Other substances, such as PCP and mephedrone, were described as being brought into Serbia from abroad. Recreational or party users mentioned the ability to purchase NPS through the DarkNet. However, procurement in this manner was rare because DarkNet browsers were uncommon, and because people feared that ordering substances to their home address could result in discovery by law enforcement. NPS were usually ordered in this fashion only if there was an option to send it to a safe address. Some respondents also reported that they obtained NPS through friends and acquaintances who either ordered these substances online with the help of someone else, or brought them in from abroad.

These methods of purchasing NPS were not without risks. In Belarus, police were a concern when buying through online channels. Law enforcement agencies sometimes created false DarkNet pages and arrested people seeking to purchase NPS. Also, if a person was in possession of a bank receipt with confirmation of payment during a police stop and search, it could be used it as justification for their arrest. It was reported that police sometimes used violence against people during arrest. After a person was taken into custody, pressure could be applied to coerce information on dealers. If a person refused to provide the information, he or she might be beaten by the police. In Georgia, almost all participants reported fear of being caught by the police. As in Belarus, law enforcement agencies created false accounts in online drug marketplaces and arrested people looking for NPS. Respondents also said that police could break into or identify a user’s account/IP address and then arrest them at the location where she or he is collecting the NPS. In Kyrgyzstan, respondents identified the risk of being arrested when collecting the NPS package from its hiding place.

### Impacts, risks and consequences of NPS use

In all countries, respondents identified a number of risks or consequences from NPS use. In Belarus, the most common negative consequences associated with NPS were psychological in nature and included paranoia, hallucinations, panic attacks, psychosis, aggression and suicidal thoughts. Negative physiological consequences mentioned by respondents included overdose, eye-gouging, motor disorders, high blood pressure and heart attacks, and well as injecting-related harms such as vein damage and endocarditis. Almost all interviewees mentioned an increase in unprotected sexual contacts. Participants also mentioned the risk of hepatitis C and HIV, particularly during long drug use sessions, during which people might inject from ten to fifteen times, sometimes sharing injecting equipment.

In Moldova, focus groups reported a number of potential harms from NPS use including paranoia, panic attacks, convulsions, overdose, unprotected sexual intercourse, physical and mental exhaustion and psychotic conditions. Respondents who used NPS also indicated that strokes and heart attacks could occur as a result of high blood pressure when using synthetic drugs. Other reported harms included unsafe injecting practices (heightened due to the frequency of injecting NPS), injecting-related vein damage and bacterial infections and risk of blood-borne virus transmission. Focus group participants also identified non-medical risks of NPS, including police pressure applied to people who use NPS in order to turn consumers into informants.

In Georgia, the main risk identified by the vast majority of respondents was overdose. This risk was heightened because people often purchased one substance that turned out to be something else. Respondents identified numerous symptoms of NPS overdose. For synthetic cannabinoids, these included lockjaw, sweating, seizures, confused consciousness and fainting. For synthetic cathinones, symptoms included hyperthermia, increased heart rate and blood pressure, coordination problems, sweating, shaking, panic attacks, hallucinations and skin (particularly the face) turning grey.

In Kazakhstan, focus groups and service providers identified various harms related to NPS use, including unsafe injecting and unprotected sexual contacts. Other harms reported included hallucinations, schizophrenia, paranoia, psychosis, panic attacks, suicidal behaviour, aggression, insomnia, encephalopathy, dehydration, injecting-related infections and abscesses, hypoventilation, heart problems and stroke and shortness of breath. In Kyrgyzstan, focus groups identified harms that included frequent injections and shared injecting equipment, lack of appetite and weight loss, unprotected sexual contacts, physical and psychological exhaustion, psychosis, paranoia, depression, heart attack and stroke and suicidal thoughts and actions. Respondents noted the appearance of more negative states of mind, such as anxiety, hallucinations and paranoia, when NPS were used in high doses and with greater frequency.

In Serbia, most of the respondents’ experience related to the use of GHB, and they identified negative reactions including vomiting, difficulty breathing and unprotected sexual contacts. Harm reduction service providers described heart palpitations and overheating as other potential negative effects, although they noted reactions varied by substance consumed. Respondents generally expressed concern about the unknown composition of substances and the overall lack of reliable information on dosage and effects. This lack of knowledge was described as applying to both consumers and sellers of NPS.

### Overdoses and response

Respondents in all countries described concerns about the dangers of NPS overdose and described various physical and psychological symptoms. In Moldova, for example, focus group participants described symptoms of a “salts” overdose. The person may initially become very agitated and confused and may faint. Their body temperature may rise and the person may experience fluctuating blood pressure, increased heart rate and pressure and pain in the chest area. Their mental state was characterised as aggressive and panicked. In the case of “salts”, death might occur either from heart failure or from cerebral oedema. If a person overdosed on “salts” and spent 20 min in that condition, it was unlikely they could be saved. All focus group participants in Moldova who used NPS knew of one or two cases of fatal “salts” overdose.

Respondents in all countries described a lack of knowledge and information on medically appropriate responses to NPS overdose, resulting in people who use drugs taking various actions. In Belarus, respondents reported using artificial respiration, providing sugar water, dousing the person with water, staying with the person to calm them down (in cases panic attack or paranoia), and even tying a person down in cases of psychosis and opening/cutting veins to let the blood flow out to lower blood pressure. Similarly in Georgia, the most common responses included artificial respiration, dousing the person with water, laying the person in recovery position, providing lemon water and staying with the person to calm them. Respondents in the other four countries all described similar types of actions in response to overdose. Respondents from the MSM community in Serbia described a common system of mutual care that had evolved in sex party settings, in which one person kept a record of the type of substances, times and doses for all the people at the party, in case any problems arose.

Despite the common experience of overdose risk and the lack of medically appropriate responses, respondents in most countries expressed a reluctance to call an ambulance due to the impact of repressive laws and policies. Only in Moldova and Georgia did people who use drugs identify calling an ambulance as a standard overdose response. Most respondents in Belarus said that although they would like to call an ambulance, in most cases they would not because of repressive drug laws. In cases of fatal overdose, police could interpret a person’s presence at the scene as evidence she or he was involved in drug distribution. Therefore, the person who called the ambulance could be prosecuted and potentially receive a prison sentence. In Kyrgyzstan, respondents were reluctant to call an ambulance due to fear of police, including fear of human rights violations and of police taking photographs and videos of people and sharing them publicly. Similarly in Serbia, people who use drugs did not typically call an ambulance as it would also summon police to the scene, and anyone present might be arrested for possession of an illegal substance. In Kazakhstan, respondents stated that if an ambulance was called, it took the NPS user straight to a psychiatric unit.

### Harm reduction services and NPS

There was a consensus among people who use drugs and service providers in all countries that the existing harm reduction services, while important, did not meet the needs people who use new psychoactives. Harm reduction services and people who use NPS noted the importance of supplementing existing harm reduction programmes and identified a number of necessary services. Commonly cited interventions included services/paraphernalia for NPS smokers and other non-injectors, provision of more diverse injecting supplies, specific information on NPS use and risks, peer-based programmes, drug checking services and training on NPS for harm reduction and health workers. The need for increased meaningful involvement of people who use drugs in designing new services was also identified.

Service providers interviewed identified the need to implement new harm reduction approaches that included peer-based interventions, case management and social/outreach support and services that respond to the needs of non-injecting (including NPS) users. Some suggested that new harm reduction approaches for non-injectors be implemented independent of existing services, which they considered unable to attract non-injecting (especially young) users to their services. At the same time, they noted that services for non-injectors should also be integrated into existing services. Harm reduction service providers identified the need for training on new harm reduction approaches and NPS-related issues.

Respondents in some countries identified policy reforms necessary to enhance the harm reduction response. In Moldova, injecting drug use was mandatory for access to a harm reduction programme, meaning people who used NPS non-intravenously were not able to register as a client, and not able to access services. The reason for this was identified as the criteria of the National Program for the Prevention and Control of HIV/AIDS and STIs, which exclusively targets prevention programmes for people who inject drugs. Harm reduction funding allocated to Moldova by the Global Fund to Fight AIDS, Tuberculosis and Malaria for 2018–2020 was directed exclusively towards injecting drug use. This resulted in organisations that provide harm reduction services in Moldova being unable to adapt their services to trends on NPS use.

Reform of punitive drug policies was also identified as necessary to improve the health and harm reduction response. In both Kazakhstan and Kyrgyzstan, fear of being placed on the Narcological Register deterred people from seeking medical help or accessing drug treatment services. The Narcological Register (also known as drug registry) registers drug users in the country and includes people diagnosed with an addiction, or are suspected of using drugs (i.e. from a positive urine test). People included on the registry are deprived of some rights, such as driving a car, working in certain jobs or getting into university for some studies. Persons listed on the register are often subject to increased scrutiny for drug use. Depending on the country, placement on the register can last from 3 to 5 years.

In Belarus, some people avoided using harm reduction services because they feared a loss of confidentiality and that their names would end up in the hands of law enforcement. In these cases, people purchased injection equipment themselves from pharmacies. In Kazakhstan, treatment protocols prohibited people who used both opioids and NPS from receiving OST treatment. However, it was common for former or current opiate users to use NPS when traditional opioids were unavailable, and poly-drug use (usually opioids mixed with any other drug) was a significant issue.

Suggestions for improving existing of harm reduction services included:

**Table:** 

Country	Harm reduction service needs identified
Belarus	Needles and syringes (different-sized needles, syringes of different colours to assist people in identifying their own syringe in circumstances where several people were using together); Disinfectants; Wound care kits; More alcohol swabs; Vending machines with safe injection kits; Condom distribution; Lights to detect veins; More information on NPS (leaflets, booklets on different NPS, risks and safer use, information on overdose and treatment); Pipes for smoking; Psychological help and support; Training for narcologists and emergency doctors on NPS (overdose, treatment, etc.); Stronger cooperation among NGOs and health services
Moldova	Pipes/mouthpieces for smoking; Sterile water to dilute salts and to prevent dehydration; Blood pressure control as a part of harm reduction programmes; Information on the use of NPS and its risks and consequences; Support groups for people who use NPS and their families; Training on NPS for harm reduction programmes, narcologists, and emergency doctors; Collaboration of harm reduction programmes with emergency medical services (ambulances); Increased funding of harm reduction programmes to allow the development and implementation of services for non-injectors
Georgia	Pipes for smoking; Foil for smoking or inhaling; Paper tubes and cards to create smooth surfaces and lines for snorting; Drug checking services; Peer-based interventions/programmes; Information on the use of NPS and its risks and consequences; Training on NPS for harm reduction programmes
Kazakhstan	More diverse drug paraphernalia (pipes for smoking, filters, sterile water, tin foil, cookers, insulin syringes, pipettes for smoking); Vitamins; Ointments and bandages; Easier access to antidepressants and sleeping pills; Safe spaces with compassionate professionals to support NPS users; Information and training on NPS-related issues, risks and harm; Rights-based training to assist people who use drugs in understanding their rights
Kyrgyzstan	Psychological and housing supports; Peer support; Information and educational materials on NPS use, risks, overdose; Paraphernalia relevant to the needs of people who use NPS, including pipes, Vaseline, condoms, lubricants; Foil; Strategies to engage hard-to-reach groups, such as young people who have never used traditional harm reduction services, and people who are purchasing drugs online; Mapping existing harm reduction services in a database of them, so that anyone who needs help or support can find all the relevant information on one website or app
Serbia	Drug checking services, particularly in places where young people congregate and socialise, such as clubs and festivals, as well as home test kits

## Discussion

This is the first study to examine the use of new psychoactive substances and the harm reduction response in Belarus, Moldova, Serbia, Kazakhstan, Kyrgyzstan and Georgia. The study is unique both in its focus on recording lived experience of people who use drugs and people who provide harm reduction services, and its cross-national nature in a region that otherwise has produced little data on NPS. Despite their cultural and political differences, and the various sub-regions in which the countries are situated, the study found remarkable similarities in patterns of NPS use and risk behaviours, markets and harm reduction gaps. The study identified several common patterns of NPS use and related harms that are worthy of discussion and further attention.

The first is the role of injecting and related risk. Injecting NPS was reported to be a significant practice in Belarus, Moldova, Georgia, Kazakhstan and Kyrgyzstan, and most likely to occur among people who have previous histories of injecting opioids. Respondents commonly described injecting NPS as characterised by short highs and frequent injections. This is consistent with research from other CEECA countries that found increased injecting episodes per day and increased sharing of injecting equipment among people who inject NPS [15, 16]. Increased frequency of injecting is linked to increased risk of blood-borne virus transmission and other injecting-related harms [30]. Serbia was the only country in the study in which injecting NPS was rare, and in which the profile of NPS use was closer to the recreational profile of NPS users in much of Western Europe [12].

The study identified a number of common reasons for choosing NPS rather than traditional illicit drugs. The primary reasons given by respondents were the lower cost of NPS and ease of accessibility. For respondents with previous histories of illicit drug use, which was the cohort most likely to inject NPS, accessibility was linked to trends in the drug market. Respondents in Belarus, Moldova, Georgia, Kazakhstan and Kyrgyzstan all described using NPS as substitutes when their traditional drug of choice (typically opioids) was not available. In general, these respondents expressed a preference for the traditional substances they used, rather than the NPS. In many cases, respondents reported they would not use NPS at all if traditional opiates were available. This is the first time this trend has been documented in these countries and is a finding consistent with several other European studies that have linked NPS injecting to decreased heroin availability [16, 31–34].

The study identified significant common gaps in the harm reduction response. Researchers identified concern about NPS overdose in all countries, which was heightened by a lack of reliable information on the effects of NPS, symptoms of NPS overdose and medically appropriate overdose responses. As a result, overdose responses were typically devised by people who use drugs themselves, who used a variety of interventions to assist the person in distress. Respondents in all countries agreed that current harm reduction services were important. However, they also identified the need to enhance and expand those services in the context of NPS. Services and supplies for non-injectors, information of NPS effects and medically appropriate overdose responses, drug checking services, peer support and training for harm reduction and health workers on new psychoactives were commonly identified. Meaningful involvement of people who use NPS was identified as a key need in developing new services and interventions.

An overarching theme that emerged in all countries was the role of punitive drug policies in driving NPS use and related harms. The negative impacts of punitive laws and policies on HIV prevention and treatment are well documented [35]. This study found crackdowns on traditional drug markets (i.e. heroin, cannabis, etc.), resulting in shrinking availability and higher prices, influenced the decision of many people who use drugs to substitute their drug of choice for cheaper and more accessible synthetic substances. However, this had the effect of driving people towards more risky or dangerous substances, the effects of which were often unknown, and the harm reduction/overdose responses unclear. For synthetic cannabinoids, people reported a variety of harmful psychological effects not typically found with the use of cannabis. For synthetic cathinones, the short-acting nature of the the euphoria led to an increased frequency of injecting, heightening the likelihood of unsafe injecting practices, injecting-related harms and transmission of blood-borne viruses. Respondents in Belarus, Moldova and Kazakhstan identified the desire to evade sanctions for positive drug tests as another reason for using NPS, again suggesting punitive drug polices as driving the new of new psychoactives.

Punitive drug polices had other negative impacts on the health response to NPS. People who use drugs in Belarus, Kazakhstan, Kyrgyzstan and Serbia expressed reluctance to call an ambulance in the case of overdose because of the likelihood of police responding with the ambulance, putting the people present at risk of arrest. This fear was further exacerbated by violent police practices reported in some countries, committed either during arrest or later while in custody to try and compel information. Respondents in Kazakhstan and Kyrgyzstan also identified the fear of being placed on Narcological Register, and the punitive impacts of such a placement, as a deterrent to calling ambulances in the case of overdose, and in accessing medical care or drug treatment services. In Belarus, some people who use drug expressed reluctance to access harm reduction services for fear of being identified by police.

## Conclusion

This study represents the first detailed, multi-country investigation of NPS use, markets and the harm reduction response in the Eurasian region. It makes an important contribution to the scarce information on the use of NPS in the six focus countries and highlights the need for enhancing the harm reduction response in the region, including the removal of punitive drug policies. It also points the way for further research on new psychoactive substances and harm reduction in the region.

## Data Availability

It is not possible to fully de-identify field notes and interview write-ups in order to fully ensure participant confidentiality. These will therefore not be made available on a public repository. However, de-identified sections of data will be made available on request, where appropriate. Full country reports are available from the Eurasian Harm Reduction Association.

## Abbreviations

ATM: Automatic teller machine
CEECA: Central and Eastern Europe and Central Asia
EHRA: Eurasian Harm Reduction Association
EK: Eliza Kurcevič
GPS: Global Positioning System
IP: Internet Protocol
MSM: Men who have sex with men
NPS: New/novel psychoactive substance(s)
OST: Opioid substitution treatment
RL: Rick Lines
